# New Approaches to Detect Biosynthetic Gene Clusters in the Environment

**DOI:** 10.3390/medicines6010032

**Published:** 2019-02-25

**Authors:** Ray Chen, Hon Lun Wong, Brendan Paul Burns

**Affiliations:** 1 School of Biotechnology and Biomolecular Sciences, The University of New South Wales, Sydney 2052, Australia; ray.chen@student.unsw.edu.au (R.C.); h.l.wong@unsw.edu.au (H.L.W.); 2 Australian Centre for Astrobiology, The University of New South Wales, Sydney 2052, Australia

**Keywords:** natural products, biosynthetic gene clusters, secondary metabolites, antiSMASH

## Abstract

Microorganisms in the environment can produce a diverse range of secondary metabolites (SM), which are also known as natural products. Bioactive SMs have been crucial in the development of antibiotics and can also act as useful compounds in the biotechnology industry. These natural products are encoded by an extensive range of biosynthetic gene clusters (BGCs). The developments in omics technologies and bioinformatic tools are contributing to a paradigm shift from traditional culturing and screening methods to bioinformatic tools and genomics to uncover BGCs that were previously unknown or transcriptionally silent. Natural product discovery using bioinformatics and omics workflow in the environment has demonstrated an extensive distribution of BGCs in various environments, such as soil, aquatic ecosystems and host microbiome environments. Computational tools provide a feasible and culture-independent route to find new secondary metabolites where traditional approaches cannot. This review will highlight some of the advances in the approaches, primarily bioinformatic, in identifying new BGCs, especially in environments where microorganisms are rarely cultured. This has allowed us to tap into the huge potential of microbial dark matter.

## 1. Introduction

Microorganisms in the environment can produce a wide range of secondary metabolites (SM). SMs are natural products with diverse chemical structures. This diversity in chemical structures enables these natural products to carry out a variety of functions. SMs can act as antibiotics, antitumor agents, cholesterol-lowering agents and so on [1]. These natural products have been critical in the development of therapeutics in medicine as it has been reported that approximately 70% of the anti-infective drugs are derived from natural products in the environment [2]. Without their discoveries, many of the therapeutics used to treat bacterial infections and diseases would not be available today.

Antimicrobial resistance is an emerging global challenge threatening future public health. Previous reports have predicted a worst-case scenario whereby the global economic impact of antimicrobial resistance will result in more than 10 million annual deaths, which is a loss of 2.0–3.5% of the world gross domestic product that is worth approximately 60–100 trillion USD by 2050 [3,4]. This growing problem emphasizes the importance of natural product discovery, particularly the search for new antimicrobials as an alternative to currently overused antibiotics. The synthesis and complexity of a natural product relies on many clustered genes playing various roles from assembly and regulation of expression [5].

Biosynthetic gene clusters (BGCs) are a physical grouping of all the genes responsible for the assembly of a SM [6]. BGCs contain genes encoding all enzymes that are required to produce a SM as well as pathway-specific regulatory genes [5]. Polyketide synthases (PKS) and non-ribosomal peptide synthases (NRPS) are two major biosynthetic systems containing multiple modules and enzymes [7]. PKS and NRPS synthesize the two major classes of SMs, which are namely polyketides and non-ribosomal peptides, respectively. PKS and NRPS are popular targets in genome mining for natural products and are well-known to synthesize a diverse range of products with beneficial applications in medicine and research, such as antibiotics, antifungals and immunosuppressants [8].

The developments in sequencing technology and readily available bioinformatic pipelines have enabled large quantities of BGCs to be mined from environmental microorganisms without having to culture them and test their bioactivity [9]. These tools also provide an incredible opportunity to elucidate the secondary metabolism properties of microbial dark matter, which is the uncultured majority of microbial diversity. Computational tools offer a feasible route to study SMs and BGCs in bacteria and are useful in generating and processing large amounts of data as seen in metagenomic studies. An extensive list of bioinformatics pipelines has been previously reviewed [10,11]. This review will highlight some notable examples, such as antiSMASH, ClustScan, NAPDOS and ClusterFinder.

Although computational tools have been useful for studying secondary metabolism from metagenomic datasets, the analysis of metagenomic data is challenging as the software requires high quality genome-resolved genomes or binned metagenomes [12]. Computational tools themselves have their own limitations, such as the reliance of databases and rules from previous knowledge, which may hinder the identification of novel BGCs as current algorithms cannot detect them. These limitations make it difficult to unlock the full chemical diversity of the environment [12].

To date, studies have screened for SMs in environments hosting large bacterial diversity and presumably large chemical diversity, such as aquatic ecosystems, soils and host microbiomes [13,14,15]. The purpose of this review is to highlight various methods used to identify BGCs in the environment and provide examples of how recent studies have explored the genetic basis for novel natural product synthesis, which have wide ranging medical and industrial applications.

## 2. Traditional Approaches in Natural Product Discovery

Prior to the advent of DNA sequencing and genomics, the search for natural products from microorganisms was conducted primarily using culture-dependent techniques in the laboratory [16]. The discovery of natural products typically involved sampling from the environment, culturing these samples and finally screening extracted products. However, it is very difficult to culture environmental microorganisms in the laboratory. The number of bacterial species that can be grown in the laboratory comprise only a small fraction of the total diversity that exists in nature [17]. Culturing microbes under different conditions was commonly used to produce and subsequently identify SMs without any knowledge of the genes and enzymes involved [18].

Biochemical assays are used primarily to screen for SMs or characterize function. Recently, the development of high throughput biochemical assays has helped to uncover more SMs. A study used thymol blue and bromothymol blue indicators to detect the pH drop in relation to sugar fermentation in *Vibrio cholerae* cultures. After screening 39,000 crude extracts, 49 were found to block fermentation and three were characterized as novel broad-spectrum antibiotics [19]. However, not all SMs can be detected and characterized using biochemical assays as some are produced at undetectable levels. Therefore, these approaches are more effective at identifying SMs that are secreted in relatively large amounts in nature and under laboratory conditions [18].

## 3. Omics Approaches for Natural Product Discovery

Traditional approaches have led to the discovery of many therapeutics that are now used today. However, natural product discovery efforts have since declined largely due to the increasing rediscovery rates of known compounds [20]. In addition, many microorganisms in the environment cannot be cultured in the laboratory, hence deterring research efforts for many years until the introduction of genomics and other omics technologies. Natural product discovery is undergoing an extensive paradigm shift, which is driven by technological developments in genomics, bioinformatics, analytical chemistry and synthetic biology [10]. Genome mining has been established as an important approach to complement bioprospecting efforts as they allow researchers to survey large datasets to determine whether the genomes of interest harbor BGCs of interest. This can be achieved before undertaking a more costly and laborious chemistry-driven approach to extract the natural product encoded by the BGC in a bacterial host. It has become possible to computationally identify thousands of BGCs in genome sequences and to systematically explore BGCs of interest for experimental characterization.

### 3.1. Metagenome Screening for BGCs Using Degenerate Primers

Degenerate primers are oligonucleotide sequences, with some positions containing more than one possible nucleotide base. This property can be used to target and amplify areas in the genome that are very similar but have slight variations [21]. This is especially useful when the same gene is to be amplified in different microbes as the same gene can vary slightly between species [8]. Degenerate primers can amplify genes of interest from the genomes of unculturable bacteria. A previous study reported that the NRPS genes associated with adenylation and thiolation domains are well-conserved in the genome, enabling degenerate primers to better target NRPS clusters in a variety of bacterial species as opposed to designing different primer sets for each species [22].

Customized primer sets were used to screen for NRPS and type I PKS (PKS-I) systems in Actinomycetes [8]. NRPS and PKS-I are known to produce a diverse range of SMs. Actinomycetes are gram-positive bacteria from the actinobacteria phylum and have been the focus for natural product discovery in previous decades due to the discovery of several antimicrobials, such as streptomycin and actinomycin, from the Actinomycetes phylum [16]. Primer sets were tested on 210 reference strains that covered the major families and 33 different genera in actinomycetes. PCR amplification of primers targeting NRPS was observed in 79.5% of strains while PCR amplification of primers targeting PKS-I was seen in 56.7% of strains [8]. The results of this study demonstrate the richness of NRPS and PKS-I-like sequences in actinomycetes, which is reflected in the diversity of antibiotics and other natural products that were previously reported in actinomycetes. Although degenerate primers can help to quantify biosynthetic capacity, they cannot be used to identify and characterize the structures of SM, which is one of the current major challenges for natural product discovery.

In a recent study, degenerate primers derived from conserved biosynthetic motifs were used to survey the ketosynthase domains from 185 soil microbiome samples [23]. BGCs encoding epoxyketone proteasome inhibitors were detected and a further analysis led to the isolation and characterization of seven epoxyketone natural products, including compounds with a unique warhead structure. Degenerate primers are useful in amplifying BGCs of interest but cannot be used to characterize a natural product. They must be used in conjunction with bioinformatic approaches with defined metagenomic tools so that natural products can be derived from metagenomic data in the environment.

### 3.2. BGC Detection and Analyses via Bioinformatic Pipelines

Many bioinformatics tools have now been developed to detect known BGCs in regular genome sequences and genome-resolved metagenomes [7]. There are also emerging tools aiming to detect novel BGCs hidden in cultured bacterial genomes and especially in environmental genome-resolved metagenomes [24].

antiSMASH, NAPDOS and ClustScan are examples of bioinformatics software that provide low novelty but high confidence in its analysis [25,26] and thus, are suitable for users looking for gene clusters of a known biosynthetic class or for surveying all detectable BGCs in single or multiple genomes for annotation purposes.

Although many bioinformatic tools can identify biosynthetic genes with high accuracy, it implies that only biosynthetic pathways with rules implemented in the software can be detected. Therefore, any pathway that may use unknown or unrelated alternative enzymes will be missed [12]. In addition, computational tools rely heavily on high quality genome-resolved metagenomes for effective and reliable outputs [12]. The quality of the sequencing data or resolved genomes from metagenomes can influence the reliability of results. Further complications regarding the analysis of metagenomic sequencing data for BGCs have been previously reviewed in more detail [10,27].

To address the limitations of identifying novel BGCs, the ClusterFinder algorithm is a recently developed software providing low confidence but high novelty analysis [24]. Predicting gene clusters from novel classes is valuable as they have the possibility of encoding molecules with new chemical scaffolds. ClusterFinder uses a hidden Markov model that switches between BGC and non-BGC analysis to look for patterns of broad gene functions encoded in a genomic region rather than searching for the presence of specific individual signature genes. This method enabled ClusterFinder to identify a large, previously unrecognized family of gene clusters that encode the biosynthesis of aryl polyenes in a wide range of bacteria from various phyla [24].

### 3.3. Expression of Transcriptionally Silent BGCs in Host Bacteria

One of the major challenges in natural product discovery is that the vast majority of BGCs are either transcriptionally silent or expressed at very low levels under standard laboratory conditions [28]. To address this issue, silent BGCs can be switched on by manipulating genetic elements embedded within BGCs [9]. Triggering BGC expression in a native host may involve artificially knocking-in a strong promoter that is located upstream of the target BGC [28]. For example, a CRISPR-Cas9 system-based promoter knock-in strategy was used to activate multiple silent BGCs in five different Streptomyces species, which led to the discovery of a novel pentangular polyketide from *Streptomyces viridochromogenes*. Activating silent BGCs in native hosts demonstrates great potential for high throughput discovery of natural products [29].

The advances in synthetic biology techniques have resulted in silent BGCs being activated in heterologous hosts [30]. Heterologous hosts can provide a significant growth advantage over native hosts and can bypass the regulatory system in the latter. This is especially useful as BGCs can be activated in unculturable microorganisms. However, one of the major problems associated with direct cloning is the low yield of positive clones, which is caused by the nonspecific targeting of random genomic fragments. Further refinement in the direct capture of gene clusters led to the discovery of taromycin A in *Streptomyces coelicolor* [31]. Moore et al. successfully extended this method to the heterologous expression of BGCs in *Bacillus subtilis* and *Escherichia coli*, which subsequently led to the discovery of a distinct group of thiotetronic acid natural products by combining this approach with targeted genome mining [32,33].

The use of chemical elicitors provide an alternative route to enhance the expression of silent BGCs [34]. This approach aims to increase the recovery of novel SMs that were previously hidden in silent BGCs both in culturable bacteria and in the unculturable majority of bacterial groups. Nodwell et al. demonstrated the use of chemical elicitor ‘CI-ARC’ to increase the yield of SM production in a collection of Actinomycetes strains [34]. The study also successfully identified a SM with activity against both bacteria and eukaryotes. This approach provides a mechanism to distinguish between SMs without knowing their biological activity, against a background of other material that is present.

### 3.4. Emerging Bioinformatic Approaches in Natural Product Discovery

The following section describes several computational perspectives that have demonstrated potential in uncovering new BGCs that encode novel bioactive SMs.

The EvoMining approach has been recently proposed as a strategy to identify new BGC classes [35]. EvoMining assumes that the genes encoding for SM enzymes have evolved from genes in primary metabolic enzymes due to duplication and divergence over time. The EvoMining software has been developed to detect divergences in the phylogenetic trees of enzymes in core pathways shared between bacterial species. This enables the software to identify enzymes that have likely been repurposed for SM biosynthesis. Using this approach, Cruz-Morales et al. identified arseno-organic metabolites in *S. coelicolor* and *Streptomyces lividans* [35]. These arseno-organic metabolites were derived from BGCs, which code for previously unknown compounds and enzymes [35]. The EvoMining approach provides a promising insight for uncovering hidden chemical diversity by incorporating evolutionary principles into genome mining.

Large-scale comparative genomic alignment has been proposed to identify new types of biosynthetic pathways. This strategy involves detecting syntenic blocks of multiple orthologous genes that are not part of the core genome of a taxon and occur in different genomic contexts in different strains and species [10]. A study using this approach successfully identified the kojic acid and oxylipin gene clusters from the accessory genome of Aspergilus species [36]. Kojic acid and oxylipin do not have any signature genes that are specific to known pathways and hence, this approach may provide users with a way to identify novel SM genes that were previously undiscovered in well-studied microorganisms.

## 4. BGC and Natural Product Mining in Different Environments

The following sections, while not exhaustive, are designed to provide some examples of work carried out in BGC ‘mining’ from different environments, specifically providing examples from soil, aquatic and host microbiomes. Noticeably, numerous studies have demonstrated that a combination of existing laboratory approaches along with the use of computational tools have provided new insights in the bioprospecting field. This is particularly crucial for studies looking to identify new natural products from microbial dark matter.

### 4.1. Natural Product Discoveries in Soil Environments

Soil environments have been reported to hold a highly diverse range of microorganisms and are known sources for antibiotics, antifungals and other natural products that are involved in bacterial communication and interaction within a given ecosystem [37,38,39]. However, many bacteria are unculturable and therefore, a vast majority of them remain understudied [40]. A global study conducted by Charlop-Powers in 2015 compared biosynthetic diversity and NRPS/PKS diversity in soil microbiomes across the globe [13]. Soil samples were collected from five continents covering different biomes. Degenerate primers that are specific to adenylation and ketosynthase domains were used for large-scale PCR. The observation of large differences in domain abundance from all except the most proximal and biome-similar samples suggests that different soil microbiomes can encode largely distinct collections of SMs.

The iChip method developed in 2010 offered a new way to isolate and cultivate unculturable bacteria in situ from many environments [41]. The miniature diffusion chambers from the iChip device allow bacterial species to be exposed to natural growth factors in the environment, hence enabling their growth and survival. Five years later, a study applying the iChip technology on soil samples led to the discovery of ‘teixobactin’, a new antibiotic that can inhibit cell wall synthesis while having undetectable antibiotic resistance in the infectious pathogens *Staphloccous aureus* and *Mycobacterium tuberculosis* [42]. The BGC giving rise to teixobactin is composed of two large NRPS-encoding genes and was identified using a homology search tool. This study demonstrates the importance for integrating novel laboratory techniques alongside computational tools as this will enable new microorganisms to be isolated, with the potential to extract new natural products and reconstruct their metabolic pathways.

A recently published study recovered hundreds of near-complete genomes from the Northern Californian grassland soil and analyzed them using antiSMASH [43]. These genomes contained newly identified members from Acidobacteria, Verrucomicobia and Gemmatimonadetes as well as the candidate phylum Rokubacteria. These members are abundant in soils but are under-represented in culture [40,43]. Members from these phyla were also previously not known to be linked to SM production but were found to encode diverse PK and NRP BGCs that were thought to have diverged from well-studied gene clusters [43]. The study demonstrates that the biosynthetic potential for phylogenetically diverse microorganisms in soils has previously been underestimated [43]. In addition, the knowledge gained in this study may provide a framework for future studies to target novel but abundant microorganisms in soils, which may represent a source for new pharmaceutical compounds.

### 4.2. Natural Product Discoveries in Aquatic Ecosystems

Aquatic ecosystems are relatively unexplored but have been reported to harbor a high diversity of microorganisms, which may reflect the biosynthetic potential of these ecosystems [14,44]. A previous study observed marine Actinomycetes as a robust source for beneficial SMs [45]. This study determined that 85% of marine bioactive compounds identified and described from 1997 to 2008 came from Streptomyces (57%) and Salinispora (28%), both of which are members of the Actinomycetes phylum. An example of a beneficial SM isolated from Actinomycetes is marizomib (salinosporamide A). Marizomib is an anticancer drug that was first isolated from *Salinispora tropica* and is currently undergoing clinical trials as it has demonstrated high cytotoxicity against breast cancer, human colon carcinoma and non-small lung cancer [46,47].

Early and recent studies have observed specific marine microorganisms, such as Roseobacter and Pseudovibrio having potential for SM production [48,49]. Members of Roseobacter have been reported to be widespread and abundant in marine environments with diverse metabolism, thus providing an opportunity to mine their genomes for potential natural products. The work from Martens et al. used degenerate primers that were specific with conserved sequence motifs to screen the strains of Roseobacter [49]. PCR products were cloned, sequenced and compared with genes of known function, revealing genes that show similarity with PKS and NRPS. Some strains also demonstrated antagonistic activity and acetylated homoserine lactone (AHL) production, which suggests that the Roseobacter clade is a potential and largely untapped source of SM.

Pseudovibrio-related bacteria have previously been isolated from marine sources as free-living and host-associated bacteria [50,51]. They have been shown to proliferate under extreme oligotrophic conditions, tolerate high heavy-metal concentrations and metabolize potentially toxic compounds [52,53,54]. The data from studies described by Romano suggest that apart from nutrient cycling, members of host-associated Pseudovibrio can provide their host with both vitamins/cofactors and protection from potential pathogens via the synthesis of antimicrobial SMs [48]. Marine environments provide an incentive for researchers to target marine microorganisms with potentially large and useful biosynthetic capacities as seen in Actinomycetes, Pseudovibrio and Roseobacter.

In a recent study, antiSMASH and NAPDOS were used to screen for SMs in recovered genome bins from Lake Stechlin, north-east Germany [14]. Of the 243 BGCs identified, 125 were classified as terpenes and represent the most abundant cluster type. Terpene products are commonly found in plants and fungi genomes but it has been recently reported that bacterial terpene synthases are distributed widely in the environment [14]. The second most abundant cluster type are bacteriocins with 35 clusters [14]. Bacteriocins are SMs belonging to a group of antimicrobial peptides, which can target closely related or unrelated strains to the bacteriocin producing bacteria [55]. Bacteriocins have been implicated as a possible alternative to currently overused antibiotics due to their diverse structure and function [55]. In addition, bacteriocins have been observed in probiotics to inhibit gastrointestinal microorganisms or pathogens [56]. A further analysis of BGCs in individual genome bins has revealed an unclassified bacterium with a PKS cluster and three associated domains belonging to the enediynes polyketides pathway. Enediynes polyketides are SMs that have been reported to demonstrate potent anticancer and antibiotic activity largely due to an enediyne core providing cytotoxic properties [57]. Their biosynthesis is also of interest as many unique chemical features were identified, providing a potent opportunity to decipher how the biosynthetic machinery can give rise to complex and unique molecules.

### 4.3. Exploring BGCs in Host Microbiome Environments

Host microbiome environments house an enormous range of bacteria that have adapted and thrived in the host’s environment. In the past few years, studies have focused on natural product discovery in the human microbiome and have provided new insight into the search for novel SMs in different environments [15,58]. Another notable example includes the microbiome in marine sponges.

The microbiome of three deep sea sponges was screened for secondary metabolite potential by using 454 pyrosequencing and degenerate primers to target sequences associated with ketosynthase domains and adenylation domains [59]. Ketosynthase and adenylation fragments were revealed to be distinct from reference sequences in the database. This demonstrates the potential for the microbiome of these marine sponges to possess a diverse range of novel SMs. The sequence analysis of the sponges studied also determined that they have genes involved in the synthesis of streptogramin, lipopeptides and glycopeptides. These bioactive compounds are known classes of antibiotics. The study also indicates that variations in the gene sequences associated with SMs may potentially lead to the identification of natural products, which may serve beneficial roles in human health [59].

A recent study utilized a combination of chemistry, metagenomics and metatranscriptomics techniques to examine how microorganisms play a key role in human health. Using 752 metagenomic samples from the National Institute of Health Human Microbiome Project, the study found human-associated bacteria housing 3,118 BGCs that encode small molecules [15]. Many of these molecules are presumably associated with beneficial properties. Among these BGCs are thiopeptide clusters encoding antibiotics, some of which have a similar structure with molecules already in clinical trials.

## 5. Conclusions

SMs have played an important role in pharmaceuticals, research and the industry. There are numerous possibilities for expanding our understanding of chemical diversity and how these metabolites function. Various studies have found an abundance of BGCs encoding known SMs and potentially undiscovered ones, which reinforces the notion that high biodiversity can lead to high chemical diversity. In the future, we should see a continuing development of bioinformatics software to address the current limitations of genome mining, analyzing metagenomic data and compound characterization. We may also see new or improved culturing and screening techniques to better explore the vastness of SMs. Many of the discoveries to date have been attributed to many omics and metaomics approaches. However, the limitations of technology and methods still need to be addressed in order to fully exploit the genome mining process and optimize the workflow from the identification of BGC to the expression of the corresponding natural product.

There is growing interest to uncover biosynthetic pathways and new natural products in the environment, particularly environments hosting a rich diversity in microorganisms, such as soils, marine ecosystems and host microbiomes. In addition, other unique microbial ecosystems that occur in often extreme environments, such as microbialites, may also be a new source of novel SM [60]. The identification of a wide range of SMs will help to provide a better understanding of how novel SMs are assembled and detected. The increasing trends and updates in bioinformatics and refinement of the metaomics workflow will complement natural product discovery efforts in the environment and may ultimately lead to an influx of novel compounds with potential applications in medicine and industries.
